# Broader phenology of pollinator activity and higher plant reproductive success in an urban habitat compared to a rural one

**DOI:** 10.1002/ece3.6794

**Published:** 2020-09-21

**Authors:** Vincent Zaninotto, Xavier Raynaud, Emmanuel Gendreau, Yvan Kraepiel, Eric Motard, Olivier Babiar, Amandine Hansart, Cécile Hignard, Isabelle Dajoz

**Affiliations:** ^1^ Sorbonne Université, CNRS, IRD, INRAE, Université de Paris, UPEC Institute of Ecology and Environmental Sciences‐Paris (iEES‐Paris) Paris France; ^2^ Paris Green Space and Environmental Department (DEVE) Paris France; ^3^ Station d'Écologie Forestière Université de Paris Fontainebleau France; ^4^ Centre de recherche en écologie expérimentale et prédictive (CEREEP‐Ecotron IleDeFrance) Département de biologie, École normale supérieure, CNRS , PSL University St‐Pierre‐les‐Nemours France

**Keywords:** flowering phenology manipulation, *Lotus corniculatus*, phenology, plant reproductive success, plant–pollinator interactions, pollinator assemblage composition, *Sinapis alba*, urban–rural gradient

## Abstract

1. Urban habitat characteristics create environmental filtering of pollinator communities. They also impact pollinating insect phenology through the presence of an urban heat island and the year‐round availability of floral resources provided by ornamental plants.
2. Here, we monitored the phenology and composition of pollinating insect communities visiting replicates of an experimental plant assemblage comprising two species, with contrasting floral traits: *Sinapis alba* and *Lotus corniculatus*, whose flowering periods were artificially extended. Plant assemblage replicates were set up over two consecutive years in two different habitats: rural and densely urbanized, within the same biogeographical region (Ile‐de‐France region, France).
3. The phenology of pollination activity, recorded from the beginning (early March) to the end (early November) of the season, differed between these two habitats. Several pollinator morphogroups (small wild bees, bumblebees, honeybees) were significantly more active on our plant sets in the urban habitat compared to the rural one, especially in early spring and autumn. This resulted in different overall reproductive success of the plant assemblage between the two habitats. Over the course of the season, reproductive success of *S. alba* was always significantly higher in the urban habitat, while reproductive success of *L. corniculatus* was significantly higher in the urban habitat only during early flowering.
4. These findings suggest different phenological adaptations to the urban habitat for different groups of pollinators. Overall, results indicate that the broadened activity period of pollinating insects recorded in the urban environment could enhance the pollination function and the reproductive success of plant communities in cities.

## Graphical abstract

We monitored the phenology and composition of pollinating insect communities visiting replicates of an experimental plant assemblage in two different habitats: rural and densely urbanized. Results indicate a broadened activity period of pollinating insects in the urban environment, which might explain the enhanced reproductive success of our focal plants in this habitat.

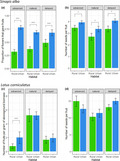

## 1 INTRODUCTION

Urbanization is one of the main and fastest‐acting drivers of land‐use changes (Grimm et al., 2008; Patacchini & Zenou, 2009), leading to strong consequences on species richness (McKinney, 2008). In dense urban habitats, pollinating insect communities are affected by habitat loss and fragmentation, contaminants, modifications of floral resources and nesting habitats, and local climate warming (Harrison & Winfree, 2015). This generates an environmental filter that can alter the composition and diversity of pollinator assemblages. While some studies have witnessed a loss of functional and taxonomic diversity in urban habitats (Deguines, Julliard, de Flores, & Fontaine, 2016; Geslin et al., 2016), others found a positive impact on pollinator diversity, especially for wild bees (Baldock et al., 2015; Fortel et al., 2014; Theodorou et al., 2017; Wenzel, Grass, Belavadi, & Tscharntke, 2020). Indeed, several characteristics of urban habitats, such as the year‐round abundance and diversity of floral resources (Baldock et al., 2019; Garbuzov, Samuelson, & Ratnieks, 2015; Stelzer, Chittka, Carlton, & Ings, 2010), and the overall warmer urban climate (Harrison & Winfree, 2015; Rizwan, Dennis, & Liu, 2007) may render them favorable for some pollinators (Hall et al., 2016), especially compared to intensive agricultural lands (Baldock et al., 2015).

Urban habitat characteristics can also impact the phenology of plant–pollinator interactions. Concerning plants, the warmer urban climate (through the presence of an urban heat island, hereafter UHI) may either advance or delay (Jochner & Menzel, 2015; Neil, Landrum, & Wu, 2010) plant flowering phenology. Moreover, the year‐round presence of ornamental plants in urban green spaces may extend the availability of floral resources for pollinating insects (Tasker, Reid, Young, Threlfall, & Latty, 2020). Individual species may display various phenological responses, ultimately causing shifts in potential interaction partners and transforming the mutualistic networks (Harrison & Winfree, 2015). Concerning pollinators, the UHI should enable them to be more active throughout the season than in rural habitats. Indeed, some recent studies report a broadening of the flight period of pollinators in the city, whereas pollinator activity tends to peak earlier in spring in seminatural habitats (Harrison, Gibbs, & Winfree, 2018; Leong, Ponisio, Kremen, Thorp, & Roderick, 2016; Luder, Knop, & Menz, 2018; Wray & Elle, 2015). This extended period of activity may also be supported by the above‐mentioned year‐round availability of floral resources in cities. Taken together, these plant and insect phenological changes should strongly impact the pollination function in urban habitats. However, there is concern that plants and pollinators might have different responses to warming, potentially leading to loss of phenological synchrony that would disrupt the pollination networks (Forrest, 2015; Memmott, Craze, Waser, & Price, 2007), although this appears to be dependent on the biodiversity level (Bartomeus et al., 2013). It has been theorized that the local adaptive responses of plant–pollinator networks to UHI effect could be considered as a small‐scale model for the larger‐scale consequences of global warming (Jochner & Menzel, 2015).

Here, we set up an all‐season monitoring of the pollination activity, pollinator assemblage composition, and the resulting pollination function, in an urban–rural paired experimental design encompassing a dense urban habitat (the city of Paris, France) and rural habitats located within the same region (Ile‐de‐France region, France). In order to standardize our monitoring from the beginning of spring to mid‐autumn, and also to simulate potential climate change‐induced modifications in the flowering phenology of plants, we used temporal transplants of an experimental plant assemblage (Morton & Rafferty, 2017), comprising two insect‐pollinated plant species native to this region. In other words, we brought plants to bloom in and out of their natural flowering period. These plant assemblages, whose flowering phenology was either “advanced” or “delayed” (in contrast to “natural”), thus played the role of plants with shifted phenological patterns. We aimed to investigate whether these out‐of‐season floral resources would find matching pollinators in the dense urban and the rural habitats investigated, and what consequences it would have on the reproductive success of the plants. Hence, these controlled plant sets can be considered as “pollinometers” (Theodorou et al., 2017), as measuring their reproductive success could be a proxy to assess the efficiency of the pollination function throughout the season between urban and rural habitats within the same region.

Our hypothesis is that, in the city, pollinator activity would show different phenological patterns that in a rural habitat. This would lead to differences in the efficiency of the pollination function and contrasting plant reproductive success over the time between these two habitats. More precisely, we expect a broadening of the pollinator flight season in the urban habitat, thus leading to more efficient early and/or late pollination, and higher overall plant reproductive success in urban habitats compared to rural ones.

To our knowledge, this is one of the few studies (Rafferty, Caradonna, Burkle, Iler, & Bronstein, 2013) that have associated an all‐season monitoring of pollinator activity to the evaluation of the pollination function, through the assessment of plant reproductive success in an urban–rural paired design.

## 2 MATERIALS AND METHODS

### 2.1 Experimental sites

Experiments were conducted over two consecutive years in four (2018) and six (2017) locations in grasslands located in dense urban habitat and forest‐dominated seminatural habitat—hereafter referred to as “rural.” All sites were located in the same biogeographical region: the Ile‐de France region that encompasses a large diversity of habitats, from the city of Paris (largest city in France) to seminatural and rural habitats (INSEE, 2015).

**FIGURE 1:**
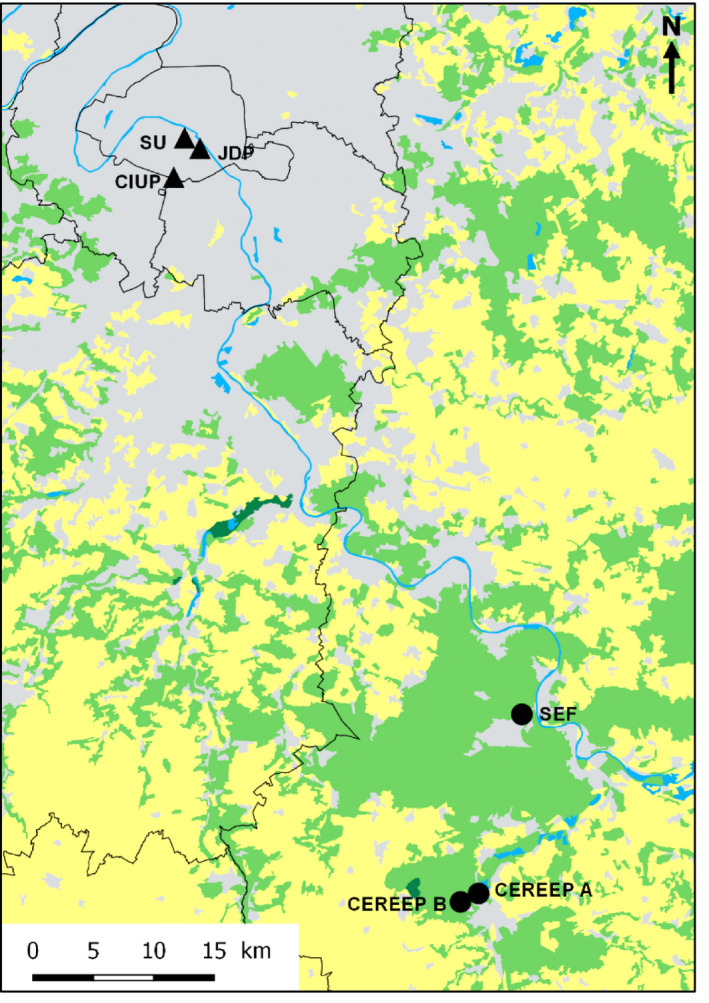
Distribution of rural (dots) and urban (triangles) sites (SEF: Station d’Ecologie Forestière of Fontainebleau‐Avon; CEREEP A and B: CEREEP‐Ecotron Ile‐de‐France; SU: Pierre et Marie Curie Campus of Sorbonne Université; JDP: Jardin des Plantes; CIUP: Cité Internationale Universitaire of Paris). Colors represent areas dominated by agricultural landscape (yellow), by seminatural habitats (green), or by impervious zones (gray). Water‐covered surfaces are represented in blue (source: European Environment Agency, Corine Land Cover, 2018)

In 2017, urban experimental sites were located in downtown Paris: Pierre et Marie Curie Campus of Sorbonne Université (SU), Jardin des Plantes (JDP), and Cité Internationale Universitaire de Paris (CIUP), whereas rural sites were all located in the Seine‐et‐Marne administrative department (50–64 km from Paris): CEREEP‐Ecotron Ile‐de‐France, with two set‐ups 1 km apart (CEREEP A and CEREEP B); and Station d’Ecologie Forestière of Fontainebleau‐Avon (SEF). In 2018, the same experimental sites were used, except for CIUP and CEREEP A. Urban sites were set in green spaces, with a combination of lawns and ornamental flower beds which do not receive any pesticide treatment. The surrounding landscapes consisted mostly of dense urban landscape and urban green spaces (Table 1, Figure 1). On the other hand, rural sites were set up in grasslands mostly surrounded by forests. These grasslands are not harvested and do not receive any chemical inputs. The SEF site is part of a forest biosphere reserve, while the two CEREEP sites are located in a large experimental ecology field station encompassing seminatural forests and grasslands. For this reason, all these sites can be considered as “seminatural,” despite their potential proximity to discontinuous suburban areas.

**TABLE 1:** Proportions of land‐use categories within a radius of 500 m around the experimental sites (SEF: Station d'Ecologie Forestière of Fontainebleau‐Avon; CEREEP A and B: CEREEP‐Ecotron Ile‐de‐France; SU: Pierre et Marie Curie Campus of Sorbonne Université; JDP: Jardin des Plantes; CIUP: Cité Internationale Universitaire of Paris) (source: European Environment Agency, Corine Land Cover, 2018)

Site		Coordinates	Continuous urban landscape	Discontinuous suburban landscape	Linear transportation infrastructures	Urban green spaces	Permanent grassland	Forests
Rural	SEF	48.4206, 2.7289	0	43	0	0	0	57
	CEREEP A	48.2867, 2.6781	0	26	8	0	2	64
	CEREEP B	48.2831, 2.6657	0	0	0	0	49	51
Urban	CIUP	48.8189, 2.3353	48	16	0	36	0	0
	JDP	48.8440, 2.3611	37	3	26	34	0	0
	SU	48.8465, 2.3587	51	0	17	32	0	0

### 2.2 Experimental setting

In each experimental site, two 1.6 × 1.2 m plots were set up side by side in a grassland area, each containing one of the two focal plant species (the Brassicaceae *Sinapis alba* and the Fabaceae *Lotus corniculatus*). *Sinapis alba* L. is an annual forb that grows along roads, in wastelands or near crops, and is considered naturalized in the Ile‐de‐France region (Lombard, 2001). It is an obligate outcrossing species (Cheng, Williams, & Zhang, 2012), the fruits of which are siliques containing up to eight seeds (Jauzein & Nawrot, 2011). On the other hand, *Lotus corniculatus* L. is a perennial, nitrogen‐fixing plant widespread in grasslands and disturbed habitats (Jones & Turkington, 1986). Native to the Ile‐de‐France region (CBNBP, 2016; Jauzein & Nawrot, 2011), this strictly entomophilous species (Pellissier, Muratet, Verfaillie, & Machon, 2012; Stephenson, 1984) bears cylindrical pods containing up to 30 seeds. No spontaneous *L. corniculatus* or *S. alba* conspecifics were found in a 100 m radius around either urban or rural site.

**FIGURE 2:**
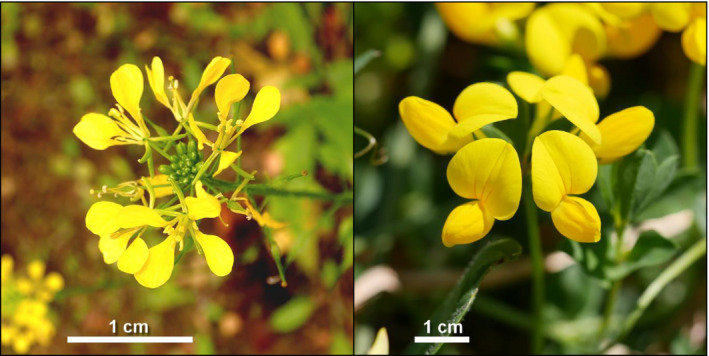
Detail of the flowers of each focal plant species: *Sinapis alba* (Brassicaceae, left) and *Lotus corniculatus* (Fabaceae, right). © Alexis Orion (CC BY 4.0), originals can be retrieved at www.inaturalist.org/photos/56174372 and www.inaturalist.org/photos/71587817. The photographs have been cropped and the scale bars added

Although they both bear yellow flowers, these two plant species were chosen for their contrasting floral morphologies, in order to attract a diverse range of pollinators: *S. alba* has flat corollas with floral rewards accessible to pollinators with short mouthparts (Fontaine, Dajoz, Meriguet, & Loreau, 2006; Geslin, Gauzens, Thébault, & Dajoz, 2013; Jones & Turkington, 1986), whereas *L. corniculatus* has deep corollas with nectar and pollen resources mainly accessible for pollinators with long mouthparts (Figure 2). Seeds of these two species (obtained from Semences du Puy, France) were germinated and grown in individual pots in a commercial potting substrate under insect‐proof greenhouse conditions (temperature: 20°C; photoperiod: 16 hr of day; 12‐cm‐diameter plastic pots filled with peat‐enriched sowing soil: 180 g.m^−3^ N, 450 g.m^−3^ P_2_O_5_, 90 g.m^−3^ K_2_O). Plants were installed in the experimental plots when flowering.

In each plot, 20 pots containing one plant of the same species were buried in four rows of five, each plant being spaced from others by 25 cm in all directions. We kept plants in their plastic pots to prevent competition for soil resources. A plastic tag was planted in each pot to individually number each plant, and all plants were watered regularly. The plots were regularly weeded to avoid interference from spontaneous plants.

Since the objective was to maintain a regular floral cover throughout the study period, and since the full flowering stage of both plant species did not exceed three weeks (V. Zaninotto, pers. obs.), the plants were renewed regularly in each plot, on the same day for all experimental sites. For both species, blooming plants were exposed to pollinators for about 20 days, before being replaced by fresh plants from the greenhouse, thus defining successive floral rounds (Table 2). At the end of each floral round, several randomly chosen plants were brought back to an insect‐proof greenhouse in order to estimate their reproductive success during the field exposure period (see fruit set and seed set measurement section).

**TABLE 2:** Summary table of flowering periods for the successive floral rounds and distribution of the floral rounds among the advanced, the natural, and the delayed flowering periods for *S. alba* and *L. corniculatus*, during both years of the experiment

Round *n*°	Period	*S. alba* flowering	*L. corniculatus* flowering
1	5 Mar.–26 Mar.	Advanced	Advanced
2	27 Mar.–17 Apr.		
3	18 Apr.–9 May		
4	10 May–31 May	Natural	Natural
5	1 Jun.–25 Jun.		
6	26 Jun.–13 Jul.		
7	22 Aug.–11 Sept.	Delayed	
8	12 Sep.–3 Oct.		Delayed
9	4 Oct.–25 Oct.		
10	26 Oct.–13 Nov.		

This design was set up during two consecutive years, in 2017 and 2018. In 2017, the study period focused on the spring season: Five 20‐day floral rounds were conducted from the beginning of March to early July. In 2018, the monitoring was extended to summer and autumn, with 10 floral rounds from early March to mid‐November, with an interruption between mid‐July and mid‐August due to the climatic conditions (severe heat waves that were harmful to the plant installations). Since the natural flowering periods of the focal plant species in the Ile‐de‐France region extend from May to July for *S. alba*, and from May to August for *L. corniculatus* (Jauzein & Nawrot, 2011), some of the flowering rounds were set before, and after, that period. Plants that were artificially brought to bloom during these rounds can be described as temporal transplants. Three phases were thus defined in the experiment (see Table 2): the *advanced* (March to April for both species), the *natural* (May to July for *S. alba*, May to August for *L. corniculatus*), and the *delayed* (August to November for *S. alba*, September to November for *L. corniculatus*) flowering periods.

### 2.3 Monitoring

During these periods, the plant–pollinator interactions on these plant plots were regularly monitored. Twice a week, all locations were monitored on the same day, in alternating order. A total of 398 five‐minute observation sessions were conducted in 2017 on each plant species, spread over 6 locations and during 4 months. A further 444 sessions were conducted in 2018 on each species, spread over 4 locations and during 7 months.

At each monitoring, the time of the day, temperature, and proportion of cloud cover were recorded. Floral display size was also qualitatively estimated at the plant level, using an index from 0 (all flowers in buds) to 3 (all visible flower buds fully opened), which provides an estimation of the quantity of floral resources in each plot. Then, for each plant species, we conducted two 5‐min observation sessions with a 10‐min break between sessions. During these sessions, all pollinator visits on experimental plants were recorded at the plant level. A visit was defined as an insect landing on a flower and inserting its mouthparts in the corolla, resulting in contact between the flower visitor and the fertile parts of the flower (stigma and/or anthers). Pollinators were identified on the fly as belonging to one of the following morphological groups: honeybees, bumblebees, large solitary bees (body length > 1 cm), small wild bees (body length < 1 cm), syrphid flies, butterflies, and beetles.

### 2.4 Fruit set and seed set measurement

At the end of each 20‐day floral round, and in all experimental sites, five *S. alba* and three *L. corniculatus* plants were randomly selected to estimate fruit set, while two plants of each species were selected to estimate seed set. Control plants were also grown in order to estimate the selfing rate and resulting fruit set and seed set of the two focal species. In 2017, one control plant per species and per round was kept in the greenhouse during its entire flowering period. In 2018, one control plant per species, per locality, and per round was set up in the field in an insect‐proof mesh cage and then brought back to the greenhouse at the end of each round. This allowed to determine a selfing rate and resulting fruit set and seed set of the two species under greenhouse conditions and under natural conditions. After each round, both plants exposed to insect visitation and control plants were kept in the insect‐proof greenhouse for an additional two weeks to allow for fruit development.

We used different methods of estimating fruit set for each plant species. For *S. alba*, all flower peduncles are still visible after the flowers have wilted, even in the absence of fruit. Therefore, on each insect‐exposed plant and each control plant, we were able to count the number of fruits produced by 10 contiguous flowers on the stem from a random starting position. For *L. corniculatus*, fruit set was not assessed in 2017 as the fruit set estimator used at the time was not appropriate for this plant species. In 2018, it was estimated by counting all fruits produced on each plant. For this same species, to take into account size differences among plants (and thus size‐related differences in floral display), plant aboveground biomass was collected individually, dried in an oven for 48 hr at 60°C, and weighted (scale precision: 1 × 10^−4^ g). For both species, seed set was estimated by counting the number of seeds contained in three fruits (when present), randomly picked on each selected insect‐exposed plant and each control plant.

### 2.5 Data analysis

All data analysis was performed using R software (R Core Team, 2019, version 3.6.1). First, visitation rates were analyzed by constructing generalized mixed effect models with the “glmmTMB” function (“glmmTMB” package, Brooks et al., 2017), which deals well with zero‐inflated data. The response variable was “Visitation rate,” defined as the number of pollinator visits per plant and per 5‐min observation session, with a negative binomial distribution to account for overdispersion. Fixed effects were the habitat (“rural” or “urban”), the flowering period (“advanced”, “normal”, and “delayed”), and their interaction, as well as the flower display size of the plant, cloud cover, the relative temperature (measured temperature relative to expected seasonal temperatures), and the year. The experimental site was included as a random effect. This model was replicated for the different morphological groups of pollinators and both plant species.

We also built generalized mixed effect models to analyze fruit set estimators. For *S. alba*, the response variable was the proportion of flowers that gave fruits, with a binomial distribution. Fixed effects were the habitat, the flowering period (“advanced”, “normal”, and “delayed”) and their interaction, as well as the year; the experimental site was again included as a random effect. For *L. corniculatus*, the response variable was the total number of fruits on the plant, with a Poisson distribution. Fixed effects were the habitat, the flowering period, and their interaction, as well as the aboveground dry mass of the plant, with the experimental site as a random effect. The same types of models were used for both plant species to analyze seed set estimators, with the number of seeds per fruit as the response variable following a Poisson distribution.

For all models, we evaluated the contribution of each factor to the model via type III Wald chi‐square tests (“ANOVA” function in “car” package, Fox & Weisberg, 2019) and performed model selection based on the AIC (“step” function, “backward” method). We also verified the absence of multicollinearity between the predictors (“check_collinearity” function from “performance” package, Lüdecke, Makowski, & Waggoner, 2020). We compared visitation rates, fruit set, and seed set between habitats within flowering periods through post hoc Tukey's tests with the “emmeans” and “contrast” functions (“emmeans” package, Lenth, 2020).

In addition, nonparametric Wilcoxon tests were carried out to compare fruit set estimators of control plants kept under insect‐proof conditions with plants exposed to pollinators, in order to estimate the rates of self‐fertilization of the two plant species. Eventually, the control plants of both species did not produce enough fruits to be able to estimate their seed set.

## 3 RESULTS

### 3.1 Pollinator visit frequencies

In 2017, over the 5 conducted floral rounds, we observed 6,364 interactions between the 8 morphological groups of floral visitors and the two plant species, with 80.4% of these visits on *S. alba* and 19.6% on *L. corniculatus*. In 2018, over the 10 conducted floral rounds, 12,096 interactions were observed in total, with 59.9% of these visits on *S. alba* and 40.1% on *L. corniculatus*.

**FIGURE 3:**
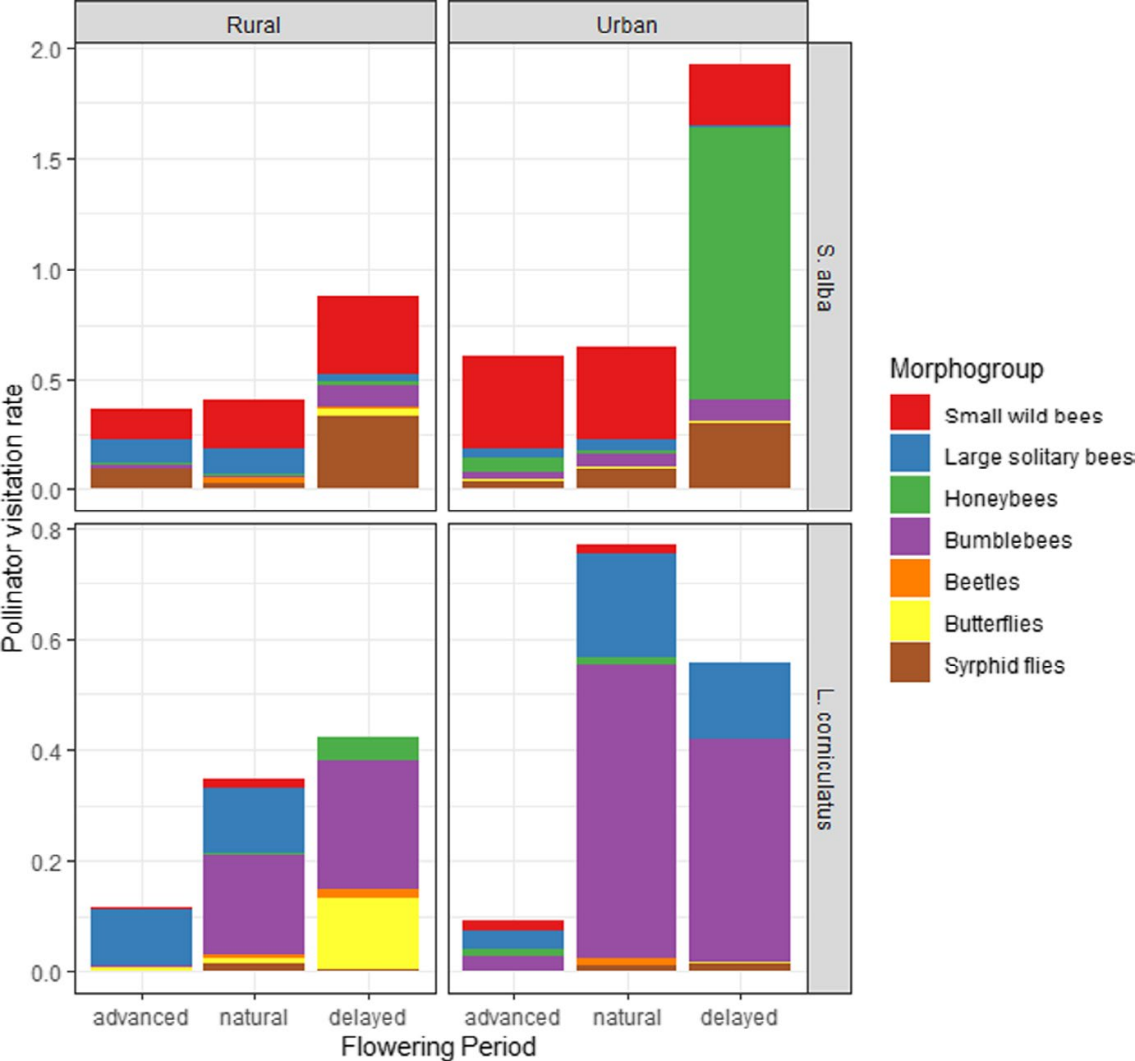
Pollinator visitation rates (mean number of visits per 5‐min session) per morphogroup (stacked) and flowering period, for the two plant species: *S. alba* (upper two graphs) and *L. corniculatus* (lower two graphs)

Over the two years of sampling, plant individuals of *S. alba* were most often visited by small wild bees (3,434 interactions, 42.5% of total interactions) and syrphid flies (1,029 interactions, 12.7% of total interactions) in the urban habitat, as well as in the rural habitat (1,828 interactions for small wild bees, 42.8% of total interactions; 955 interactions for syrphid flies, 22.3% of total interactions) (see Figure 3 for a graphical representation of mean visitation rates). However, large solitary bees visited *S. alba* more frequently in rural sites (778 interactions, 18.2% of total interactions) compared to urban sites (295 interactions, 3.65% of total interactions), while bumblebee visits remained rare in both rural (253 interactions, 5.92% of total interactions) and urban sites (510 interactions, 6.31% of total interactions). During the delayed flowering period, domestic honeybees generated a peak of visits in the city (2,349 interactions, 63.5% of interactions observed during this flowering period), whereas they were almost absent in rural sites (22 interactions, 1.40% of interactions during this flowering period).

On the other hand, during the 2 years of sampling, visits on *L. corniculatus* were largely dominated by bumblebees in both habitats (2,739 interactions, 67.2% of interactions in urban habitat; 945 interactions, 46.7% of interactions in rural habitat), and to a lesser extent by large solitary bees (1,005 interactions, 24.7% of interactions in urban habitat; 624 interactions, 30.8% of interactions in rural habitat) (see Figure 3). Butterfly visits mainly occurred in the delayed flowering period in the rural habitat (159 interactions, 30.3% of interactions during this flowering period).

As we could expect, our models reveal that pollinator visitation rates to a plant are strongly associated with the floral display of that plant, but also with the relative temperature at the time of observation. On both plant species, we also observed differences between the two habitats that varied throughout the season, as evidenced by the terms “Habitat,” “Flowering Period,” and their interaction (Table 3).

**TABLE 3:** Summary table of the best‐fitting glmm models of visitation rates on the two plant species, for main pollinators

Plant	Response var.	AIC	Effects	*df*	*χ* ^2^	*p*‐Value	Estimates
*S. alba*	All pollinators’ visitation rate	30,717	Floral display	1	2008.4	<2e−16	**1.19** ± 0.03
			Relative temperature	1	190.7	<2e−16	**0.30** ± 0.02
			Habitat	1	12.3	0.00046	NS (urb.)
			Flowering_period	2	428.7	<2e−16	−**0.26** ± 0.07 (adv.)|**0.60** ± 0.08 (del.)
			Habitat × Flowering_period	2	27.6	1.0e−06	**0.18** ± 0.09 (urb. adv.)|**0.56** ± 0.11 (urb. del.)
	Small wild bees’ visitation rate	17,663	Floral display	1	881.7	<2e−16	**1.14** ± 0.04
			Relative temperature	1	319.6	<2e−16	**0.64** ± 0.04
			Habitat	1	3.2	0.073	NS (urb.)
			Flowering_period	2	29.6	3.7e−07	−**0.73** ± 0.11 (adv.)|NS (del.)
			Habitat × Flowering_period	2	57.9	2.7e−13	**0.71** ± 0.14 (urb. adv.)| **−0.45** ± 0.16 (urb. del.)
	Syrphid flies’ visitation rate	8,918	Floral display	1	321.0	<2e−16	**0.97** ± 0.05
			Habitat	1	1.2	0.27	**0.85** ± 0.17 (urb.)
			Flowering_period	2	374.5	<2e−16	**0.79** ± 0.17 (adv.)|**2.19** ± 0.17 (del.)
			Habitat × Flowering_period	2	70.1	6.1e−16	**−1.84** ± 0.22 (urb. adv.)| **−0.99** ± 0.22 (urb. del.)
*L. corniculatus*	All pollinators’ visitation rate	15,293	Floral display	1	674.6	<2e−16	**1.09** ± 0.04
			Relative temperature	1	164.7	<2e−16	**0.50** ± 0.04
			Year	1	116.8	<2e−16	**1.02** ± 0.10 (2018)
			Habitat	1	2.8	0.092	**1.01** ± 0.34 (urb.)
			Flowering_period	2	394.9	<2e−16	**−1.38** ± 0.14 (adv.) | **−0.56** ± 0.14 (del.)
			Habitat × Flowering_period	2	40.4	1.7e−09	**−1.21** ± 0.19 (urb. adv.) | NS (urb. del.)
	Bumblebees’ visitation rate	8,882	Floral display	1	350.0	<2e−16	**1.24** ± 0.07
			Relative temperature	1	46.8	7.9e−12	**0.39** ± 0.06
			Year	1	50.0	1.6e−12	**1.00** ± 0.14 (2018)
			Habitat	1	17.8	2.4e−05	**1.32** ± 0.30 (urb.)
			Flowering_period	2	221.2	<2e−16	**−4.44** ± 0.47 (adv.) | NS (del.)
			Habitat x Flowering_period	2	8.0	0.019	**0.92** ± 0.52 (urb. adv.) | −**0.52** ± 0.27 (urb. del.)
	Large solitary bees’ visitation rate	6,186	Floral display	1	194.5	<2e−16	**0.98** ± 0.07
			Relative temperature	1	78.0	<2e−16	**0.65** ± 0.07
			Year	1	127.8	<2e−16	**1.87** ± 0.17 (2018)
			Habitat	1	0.2	0.68	NS (urb.)
			Flowering_period	2	88.5	<2e−16	**−1.25** ± 0.15 (adv.) | **−1.11** ± 0.16 (del.)

Note

For each term, chi‐square and *p*‐value of the type III Wald chi‐square tests are presented, as well as the estimates of each coefficient (±*SE*) (“urb.” = urban; “adv.” = advanced; “del.” = delayed; rural habitat, normal flowering period, and year 2017 were taken as references).

**FIGURE 4:**
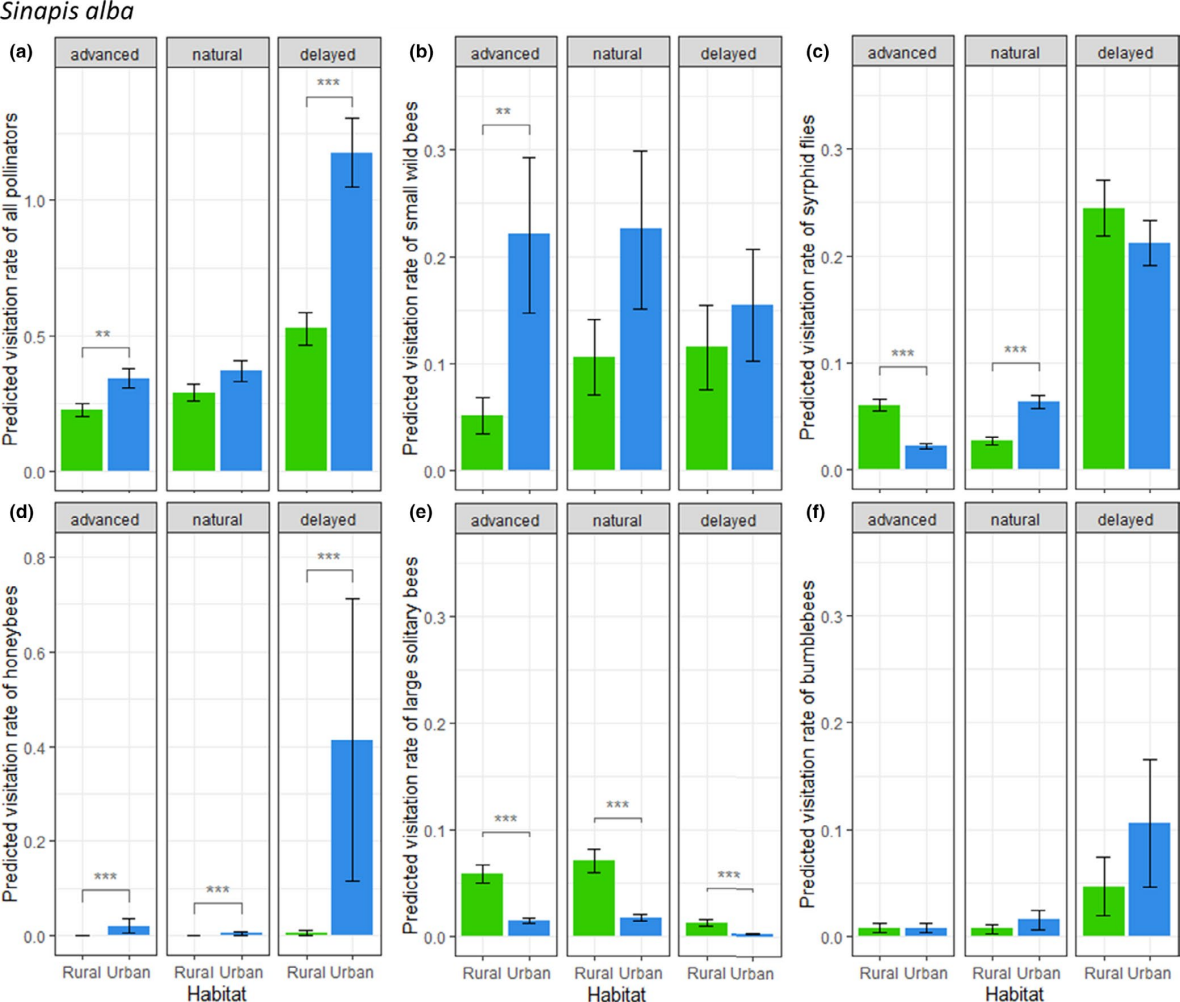
Predicted pollinator visitation rates (number of visits per 5‐min session) on *Sinapis alba*, per floral round, for all pollinators combined (a) and major pollinator morphogroups: (b) small wild bees, (c) syrphid flies, (d) honeybees, (e) large solitary bees, (f) bumblebees. Bars represent estimated marginal means ± *SE* (green = rural; blue = urban). Stars represent significance levels from Tukey's post hoc tests

Overall, pollinator visits on *S. alba* were not restricted to the natural flowering phenology of the plant in both habitats (Figure 4a). Small wild bees and large solitary bees visited this plant species from the beginning of the advanced‐flowering period (Figure 4b,e), while syrphid flies, domesticated honeybees, and bumblebees performed the most visits during the delayed blossoming period (Figure 4c,d,f). First, overall visitation rates on *S. alba* were higher in the urban habitat during the advanced‐flowering period (*t* = −2.87, *df* = 16,830, *p* = .0041, Figure 4a). At that time, this difference seemed to rely on higher visitation rates of small wild bees in the urban habitat (*t* = −3.09, *df* = 16,830, *p* = .0020, Figure 4b), while those of syrphid flies (*t* = 6.80, *df* = 16,831, *p* < .0001), and large solitary bees (*t* = 7.89, *df* = 16,832, *p* < .0001), were significantly lower (Figure 4c,e). Then, during the natural flowering period of *S. alba*, lower visitation rates of large solitary bees in the urban habitat seemed to be compensated by higher visitation rates of syrphid flies. Therefore, there was no significant difference between the overall visitation rates in the two habitats (*t *= −1.63, *df* = 16,830, *p* = .10). Finally, during the delayed flowering period, since there was a surge in domestic honeybees’ visits in the urban habitat (Figures 3 and 4d)—while syrphid fly visitation rates increased substantially in both habitats (Table 3; Figure 4c)—overall visitation rates again became significantly higher (*t* = −5.07, *df* = 16,830, *p* < .0001) in the urban habitat than in the rural one.

**FIGURE 5:**
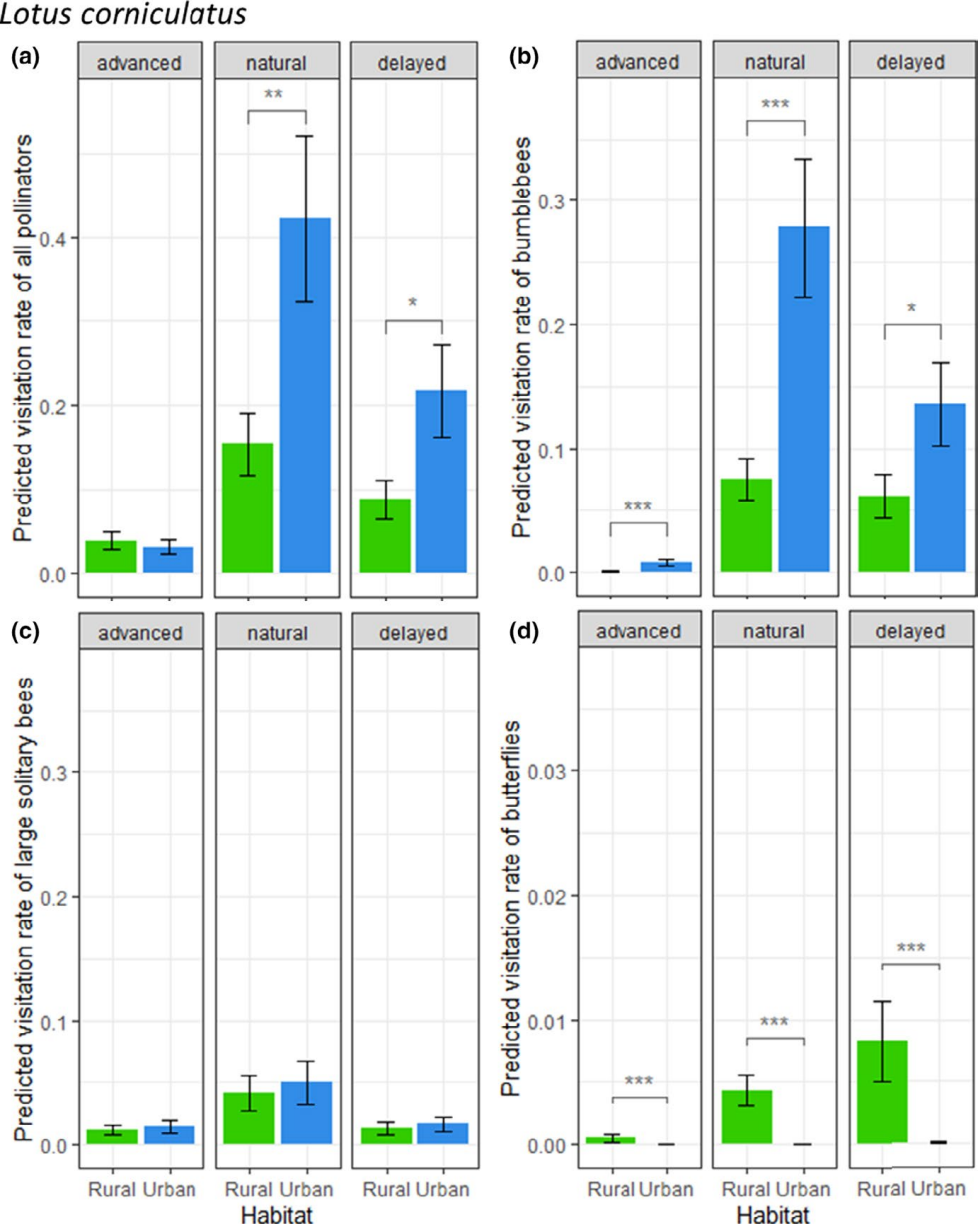
Predicted pollinator visitation rates (number of visits per 5‐min session) on *Lotus corniculatus*, per floral round, for all pollinators combined (a) and major pollinator morphogroups: (b) bumblebees, (c) large solitary bees, (d) butterflies. Bars represent estimated marginal means ± *SE* (green = rural; blue = urban). Stars represent significance levels from Tukey's post hoc tests

Pollinator visits on *L. corniculatus* were more restricted by the natural flowering period of the plant (Table 3; Figure 5a), with overall more visits during this natural phenology period compared to the advanced and delayed flowering periods. During this period, bumblebee and large solitary bee visits were the most frequent (Figure 5b,c). Overall, pollinator visits were significantly more frequent in the urban habitat than in the rural one during the natural (*t* = −3.01, *df* = 14,589, *p* = .0026) and delayed (*t* = −2.47, *df* = 14,589, *p* = .014) flowering periods (Figure 5a). This was mainly driven by bumblebee visitation rates which were significantly higher in the urban habitat for all flowering periods (Figure 5b). This might have been compensated in terms of visitation rates by other pollinators in rural sites during the advanced flowering. Yet, the other main visitors of *L. corniculatus*, large solitary bees, did not show significantly different visitation rates between habitats during any flowering period (Figure 5c). On a smaller scale, butterfly visitation rates were always significantly higher in the rural habitat and increased during the delayed flowering period, albeit remaining scarce throughout the season (Figure 5d).

### 3.2 Plant reproductive success

**FIGURE 6:**
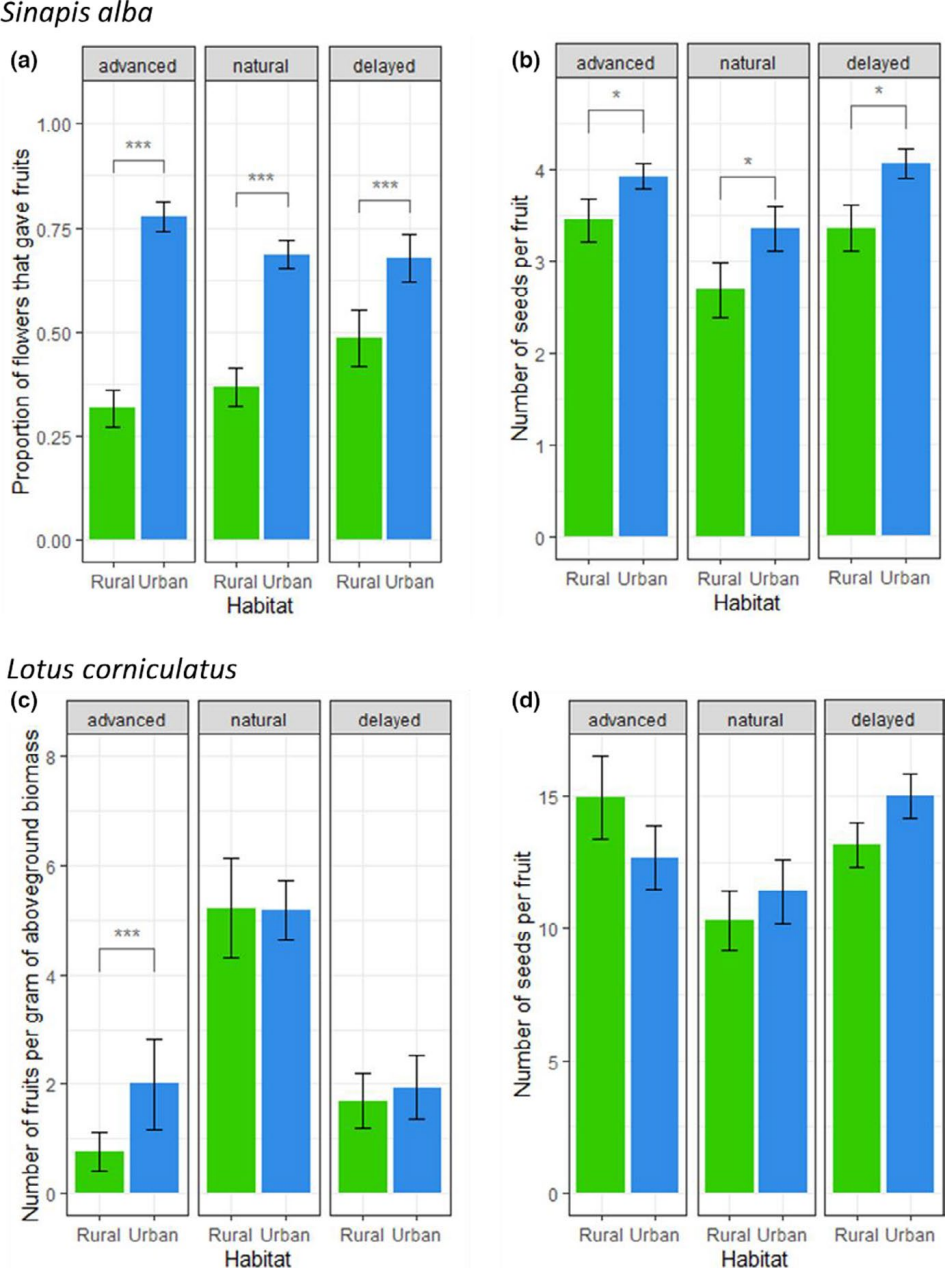
Reproductive success estimators, per flowering period, and for the two plant species: (a) and (b), respectively, fruit set and seed set rates of *S. alba;* (c and d), respectively, fruit set and seed set rates of *L. corniculatus*. Bars represent mean value ± *SE*. Stars represent significance levels from Tukey's post hoc tests

In the urban habitat, fruit set rate of *S. alba* remained elevated for the three experimental phases and was not restricted to the natural flowering time (Table 4; Figure 6a). In particular, the fruit set rate during the advanced period was already as high as than during the natural flowering period (mean percentage of flowers that gave fruits ± *SE*: advanced period, 78 ± 3.0%; natural period, 69 ± 3.0%; delayed period, 68 ± 6.0%). In contrast, rural fruit set rates were always significantly lower (Figure 6a; *df* = 352, advanced period: *t* = −10.2, *p* < .0001; natural period: *t* = −6.81, *p* < .0001; delayed period, *t* = −4.22, *p* < .0001), but they seemed to slowly increase throughout the season (advanced period, 32 ± 4.0%; natural period, 37 ± 5.0%; delayed period, 49 ± 7.0%). Last, mean fruit set rates of the control plants were significantly lower than mean fruit set rate of plants exposed to pollinators (6.0 ± 2.0% and 3.0 ± 1.0% of fruit set for 2017 and 2018 controls, respectively; no significant difference between these two values, whereas mean overall fruit set of plants exposed to pollinators in both years was 55 ± 2.0% SE, W = 1702 *p* = 1.4e−13). This indicates that differences in fruit set between habitats are not due to higher selfing rates because of differences in other biotic or abiotic characteristics of the environment. Not only was the fruit set rate of *S. alba* higher in the urban habitat, but the fruits also contained more seeds during each of the periods studied (Figure 6b; *df* = 259, for each period: *t* = −2.56, *p* = .011). This resulted in an overall higher reproductive success of the plant in the urban habitat than in the rural habitat.

For *L. corniculatus*, fruit set showed the same dynamics as pollinator visit frequencies and was consequently higher during the natural flowering period in both habitats (Table 4; Figure 6c). However, this fruit set was significantly higher in the urban than in the rural habitat during the advanced‐flowering period (*t* = −4.07, *df* = 68, *p* = .0001), though well below the value during the natural flowering time. Last, mean fruit set rates of the two types of control plants were significantly lower than mean fruit set rate of plants exposed to pollinators (no fruit has ever been observed on 2017 or 2018 controls, whereas mean overall fruit set of plants exposed to pollinators in both years was 3.11 ± 0.35 fruits per gram of dry aboveground biomass, W = 260 *p* = 1.5e−14). This also strongly suggests that differences in fruit set between habitats are not due to higher selfing rates because of differences in other biotic or abiotic characteristics of the environment. As for seed set rates, there seemed to be no difference between the two habitats throughout the season (Figure 6d).

**TABLE 4:** Summary table of the best‐fitting glmm models of fruit set and seed set of the two plant species

Plant	Response var.	AIC	Effects	*df*	*χ* ^2^	*p*‐Value	Estimates
*S. alba*	Proportion of flowers that gave fruits	2,793	Year	1	31.1	2.5e−08	**0.51** ± 0.09 (2018)
			Habitat	1	65.0	7.3e−16	**1.37** ± 0.20 (urb.)
			Flowering_period	2	3.6	0.17	−**0.28** ± 0.12 (adv.)|NS (del.)
			Habitat × Flowering_period	2	36.0	1.5e−08	**0.73** ± 0.17 (urb. adv.)|−**0.39** ± 0.20 (urb. del.)
	Number of seeds per fruit	999	Habitat	1	6.6	0.011	**0.18** ± 0.07 (urb.)
			Flowering_period	2	7.6	0.022	**0.19** ± 0.08 (adv.)|**0.21** ± 0.08 (del.)
*L. corniculatus*	Number of fruits per plant	2,369	Dry mass of the plant	1	138.8	<2e−16	**0.31** ± 0.03
			Habitat	1	4.4	0.036	NS (urb.)
			Flowering_period	2	632.2	<2e−16	−**1.84** ± 0.10 (adv.)|−**0.97** ± 0.08 (del.)
			Habitat × Flowering_period	2	77.6	<2e−16	**0.92** ± 0.10 (urb. adv.)|**0.17** ± 0.10 (urb. del.)
	Number of seeds per fruit	1,233	Habitat	1	0.3	0.59	NS (urb.)
			Flowering_period	2	26.0	2.2e−06	**0.35** ± 0.10 (adv.)|**0.28** ± 0.08 (del.)
			Habitat × Flowering_period	2	5.9	0.052	**−0.25** ± 0.13 (urb. adv.)|NS (urb. del.)

Note

For each term, chi‐square and *p*‐value of the type III Wald chi‐square tests are presented, as well as the estimates of each coefficient (±*SE*) (“urb.” = urban; “adv.” = advanced; “del.” = delayed; rural habitat, normal flowering period, and year 2017 were taken as references).

## 4 DISCUSSION

In this study, we used an experimental approach to compare the response of pollinator assemblages and their activity between urban and rural sites within the same geographical region, when confronted with a controlled plant assemblage with constant flowering throughout the season. Our results show that the phenology of pollination activity differed between the two habitats, with several pollinator morphogroups (small wild solitary bees, bumblebees, honeybees) being significantly more active on the plant assemblage in the urban habitat compared to the rural one, especially during the advanced and the delayed flowering of this plant assemblage. This resulted in contrasted reproductive success of the plants between the two habitats, with an overall reproductive success higher in the urban habitat due to the broadening of the pollinator activity season in this habitat compared to the rural one.

The phenology of visits on *S. alba* did not seem to be restricted to the natural flowering period of the plant. In particular, in the urban habitat, abundant early visits of small wild bees and an intense honeybee activity late in the season might be responsible for the high and stable measured fruit set and seed set rates of the plants, beyond the range of natural flowering period.

In contrast, *L. corniculatus* visits were predominantly carried out by bumblebees, whose visitation rates were more limited to the natural flowering period of the plant. As a result, fruit set rates, and by extension reproductive success of the plant, were more restricted to this period than in *S. alba*. This can be associated with a higher degree of specialization in the pollination ecology of *L. corniculatus*, with deep and hard‐to‐reach floral resources that are nevertheless accessible to bumblebees (Figure 2). Since these pollinators seem to be determinant to the pollination of *L. corniculatus* (Fontaine et al., 2006; Jones & Turkington, 1986), the synchronization between the natural flowering period of this plant and the activity of the bumblebees was expected. Before that, during the advanced blossom, we registered greater bumblebee activity in urban sites, though visits remained scarce in both habitats. This may explain why plants achieved a better fruit set in urban sites at that time, which could result in a better reproductive success.

Previous recent work (Harrison et al., 2018; Leong et al., 2016; Wray & Elle, 2015) highlighted different phenological patterns of bee abundance and diversity in urban versus seminatural habitats. Bee pollinating peak was reached in early spring in the forest (natural) habitat, whereas it was delayed to mid‐summer in the city (late summer in arable land for Leong et al., 2016). Both Harrison et al. (2018) and Wray and Elle (2015) found differences in trait representation linked with phenology among forest and city pollinator communities: In an urban habitat, they recorded more bees in general, with later emergence time and/or longer flight periods. Furthermore, Theodorou et al. (2020) also found that Hymenoptera diversity was negatively affected by the presence of arable/agricultural lands, thus leading to a higher diversity in the city. Here, we indeed observed a higher abundance of pollinator visits during late season in the urban habitat. This was mainly driven by the late abundance of domestic honeybees and bumblebees. However, we also observed a higher activity of small wild bees on *S. alba* and bumblebees on *L. corniculatus* in the city early in the season. Overall, this suggests different phenological adaptations to the urban habitat for different groups of pollinators.

As for syrphid flies, few studies are available. Luder et al. (2018) showed that syrphid flies are less abundant in the city than in the rural habitat, but their phenology seemed broader in the city, with an earlier appearance and a later peak. Here, we did not observe a similar phenological pattern. Hoverflies on *S. alba* showed more pronounced activity in rural sites during the advanced‐flowering period, but the contrary was found in the urban habitat during the natural flowering period. Last, during the delayed flowering period, hoverfly activity strongly increased in both the urban and the rural habitats, although no significant difference was detected between the two habitats.

Here, the observed urban phenological patterns might arise from different, although nonexclusive, processes. It might be the result of a plastic adaptation to a broader phenology in a warm environment with little temporal limitation of floral resources. Such plastic adaptation of phenology could happen at the species level. Pollinators would have to shift their emergence date or extend their flight period. Multivoltine species such as bumblebees could also benefit from a longer favorable period by producing additional generations during the year (Stelzer et al., 2010).

At the community level, the broad urban phenological pattern could also be a consequence of an environmental filtering of pollinator species in favor of generalist species (Geslin et al., 2013; Wray & Elle, 2015). As these generalist species have particularly broad phenologies, the resulting urban assemblage would have a longer flight period. Besides, the environmental filter in the city could lead to the replacement of species by others whose traits better match the phenology of plants in the urban habitat (Banaszak‐Cibicka et al., 2018). Previous studies showed this seems to be the case with small bee species of the genus *Lasioglossum* (Geslin et al., 2016). Species replacement may also be artificially enhanced by the introduction of managed honeybee colonies in the city, as the different pollinator morphogroups investigated showed variable responses to increased apiary densities (Ropars, Dajoz, Fontaine, Muratet, & Geslin, 2019).

It is very unlikely that the observed differences in fruit set and seed set between habitats were due to varying selfing rates between localities or between habitats. Furthermore, *S. alba* is an obligate outcrossing species (Olsson, 1960) and such is also the case for *L. corniculatus* (Ollerton & Lack, 1998). Here, we used three proxies of reproductive success (percentage of flowers that set fruits for *S. alba*, fruit production per unit of aboveground biomass for *L. corniculatus,* and number of seeds per fruit for both plants) to account for the influence of different pollinator assemblages and phenologies on the reproductive success of the experimental plant assemblage. However, other factors are involved in this reproductive success that might have various impacts outside the natural phenology ranges of plants. For example, Parsche, Fründ, and Tscharntke (2011) found that advanced flowering strongly enhanced reproductive success of a close species to *S. alba*, *Sinapis arvensis*. Although advanced‐flowering plants were less visited by pollinators, they also less suffered from pollen grazing beetles. Parsche et al. thus hypothesized that enhanced reproductive success during this advanced flowering may result from a trade‐off between a weaker pollination and an escape from pollen grazing. We found a similar trend concerning the fruit set and the seed set of *S. alba*, which were elevated during the advanced‐flowering period, especially in the city. Taking into account the impact of pollen grazing beetles on fruit set might provide another hypothesis for the weak fruit set of *S. alba* in the rural habitat: these plants might have suffered more from this grazing pressure than the urban ones. Indeed, the intensity of plant damage caused by pollen beetle herbivory was found to be positively associated with the proportion of crops in the landscape (Thies, Steffan‐Dewenter, & Tscharntke, 2003), and in our case, agricultural landscapes were more common in the vicinity of the rural sites (mean share of agricultural lands in a 5 km radius was, respectively, 23% and 0% around rural and urban sites, Figure 1).

In turn, Theodorou et al. (2020) also witnessed a higher seed set of their “pollinometer” species (*Trifolium pratense*) in urban habitats, apparently driven by high visitation rates of *Bombus sp* and domestic honeybees. This resonates with the high fruit set and seed set achieved in our urban habitat by *S. alba* and *L. corniculatus*. The same morphological groups of pollinators seemed involved here: respectively, domestic honeybees on *S. alba* during delayed flowering; and bumblebees on *L. corniculatus* during natural, delayed, and to a lesser extent advanced flowering.

On the other hand, Pellissier et al. (2012), while monitoring reproductive success of *Lotus corniculatus* along an urbanization gradient, observed a greater fruit set in suburban areas than in dense urban sites. Since their study was conducted during the natural flowering period of the plant, this result is not at odds with our present work: Here, we did not observe a significant difference in fruit set between urban and rural sites during the natural flowering period of the plant.

Overall, our results suggest that a flowering phenology broadening might be beneficial to *S. alba*, as elevated reproductive success rates were not limited to its natural flowering time. This positive impact of phenological broadening on reproductive success was less strong for *L. corniculatus*, even though it was detected in the urban habitat where bumblebees displayed a longer, and especially an earlier starting flight period. Besides, this plant species needs strict photoperiod conditions to initiate flowering (Steiner, 2002).

Here, we witnessed different phenological patterns of pollinator activity between an urban and a rural habitat. Still, we cannot explain the underlying causes of these differences with our experimental setting alone. The two habitats also differ by several factors, among them by their temperature. Indeed, through meteorological data we detected an UHI effect, with a mean difference of about 2°C in daily minimal temperatures between our urban and rural habitats (Figure S1). Adaptation to UHI might contribute to shape the phenology of the pollinator community in the city, with earlier in the season activity of pollinator morphogroups such as small wild bees and bumblebees (Stelzer et al., 2010). Besides, other phenomena might be involved. The temporal availability of floral resources differs substantially between the two habitats, not only because of advanced phenology of spontaneous plants in response to UHI. Thus, we cannot exclude a “honeypot effect” (Theodorou et al., 2020) of our experimental plant assemblage that might represent an important trophic resource in an otherwise resource‐poor environment, especially during early and late season, and even more during droughts. Indeed, water‐stressed plants were shown to provide less nectar sugar content per flower and consequently attract fewer flower visitors (Descamps, Quinet, Baijot, & Jacquemart, 2018). The “honeypot effect” might have concentrated the local urban pollinator community on our plants, thus increasing visitation rates and ultimately reproductive success. This phenomenon may be just as intense in the rural habitat, where proximity to massively flowering crops (Figure 1) could induce strong variations in the availability of floral resources, with alternating high‐ and low‐food supply periods (Requier et al., 2015), whereas flower resources are more stable all‐year‐round in the city (Tasker et al., 2020) thanks to the presence of ornamental plants. Indeed, in the city, abundant managed flowering plants are set in green spaces, private gardens, balconies. Though ornamental plant species attractiveness to pollinators is highly variable, they are suspected to lessen temporal resource limitations for pollinators (Garbuzov et al., 2015).

We found differences in pollinator assemblages visiting the two focal plant species used in our experimental design. This emphasizes the need to carry such experimental approaches on several plant species with contrasting floral morphologies. Indeed, it is necessary to monitor the responses of a large range of pollinator groups—with various mouthparts morphologies enabling them to visit contrasted corolla shapes—in order to assess the response of the pollination function to plant phenological changes within and among environments. Here, the assemblage of pollinators we witnessed was shaped by our choice of plant models and also by the timing of observation. Hence, it is not an exhaustive survey of all the pollinators present in the two habitats studied.

The high temporal resolution of our experiment, with two weekly monitoring sessions spanned over several months, together with the high level of maintenance required to set up the plant assemblage in the different localities surveyed, made it difficult to multiply geographical replicates. Overall, we are aware that future research on the response of the pollination function to phenological and habitat changes would benefit from a wider set of geographical replicates, especially if they could encompass an urban–rural gradient (Fisogni et al., 2020).

## CONFLICT OF INTEREST

None declared.

## AUTHOR CONTRIBUTION

**Vincent Zaninotto:** Conceptualization (equal); Data curation (lead); Formal analysis (lead); Investigation (lead); Methodology (lead); Project administration (equal); Visualization (lead); Writing‐original draft (lead); Writing‐review & editing (lead). **Xavier Raynaud:** Conceptualization (equal); Methodology (supporting); Supervision (equal); Validation (equal); Writing‐review & editing (equal). **Emmanuel Gendreau:** Conceptualization (equal); Methodology (supporting); Supervision (equal); Validation (equal); Writing‐review & editing (equal). **Yvan Kraepiel:** Conceptualization (equal); Methodology (supporting); Supervision (equal); Validation (equal); Writing‐review & editing (equal). **Eric Motard:** Investigation (equal); Methodology (equal); Supervision (equal). **Olivier Babiar:** Investigation (equal); Methodology (equal); Resources (equal). **Amandine Hansart:** Investigation (equal); Methodology (equal); Resources (equal); Writing‐review & editing (supporting). **Cécile Hignard:** Investigation (equal); Methodology (equal); Resources (equal). **Isabelle Dajoz:** Conceptualization (equal); Funding acquisition (lead); Investigation (equal); Methodology (equal); Project administration (equal); Resources (equal); Supervision (lead); Validation (equal); Writing‐review & editing (equal).

## Supporting information

Figure S1

## Data Availability

All data are archived in the publicly accessible repository Zenodo, within the “iEES‐Paris OpenData” community: https://doi.org/10.5281/zenodo.3993031.
