# Supplementary material for: Broader phenology of pollinator activity and higher plant reproductive success in an urban habitat compared to a rural one
Source: Ecol Evol. 2020 Sep 21;10(20):11607–21. doi: 10.1002/ece3.6794 (PMC7593137; doi:10.1002/ece3.6794)
Supplement: Supplementary file 1 — Figure S1 [file ECE3-10-11607-s001.docx]

## **SUPPLEMENTARY FIGURES**


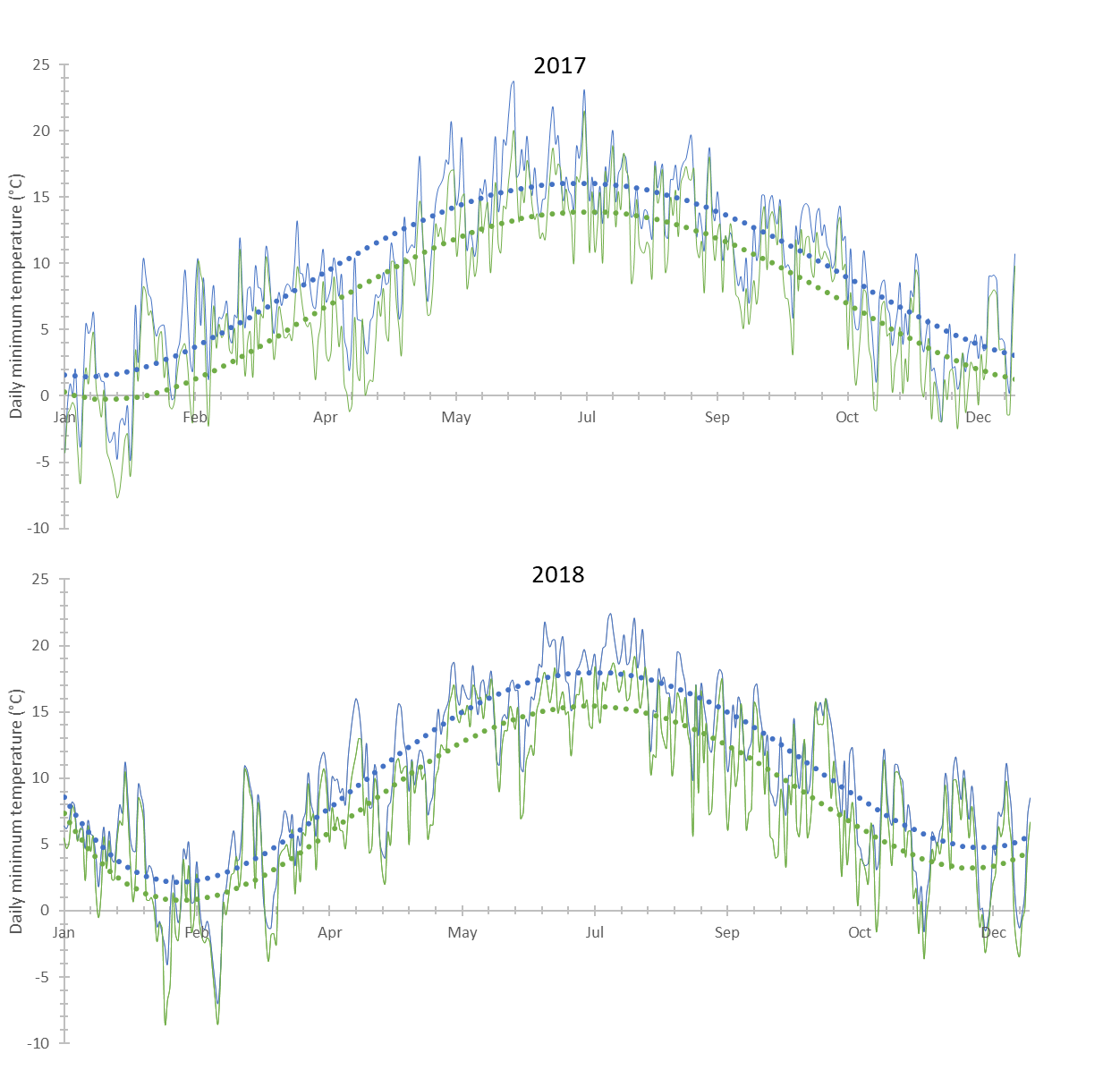
**Sup. Figure 1 – Daily minimum temperatures in urban (blue) and rural (green) habitat during both years of monitoring. Dotted lines represent polynomial fitted curves. (RADOME network public data, ‘Melun-Villaroche’ and ‘Paris-Montsouris’ stations, available at: https://www.meteo60.fr/stations-releves/station-mois)**
